# Alterations in Mitochondrial DNA in Corneal Fibroblast and Myofibroblast Post Injury

**DOI:** 10.1167/iovs.67.1.36

**Published:** 2026-01-16

**Authors:** Nishant R. Sinha, Alexandria C. Hofmann, Laila A. Suleiman, Maxwell T. Jeffrey, Rajnish Kumar, Rajiv R. Mohan

**Affiliations:** 1 Harry S. Truman Memorial Veterans’ Hospital, Columbia, Missouri, United States; 2 Department of Veterinary Medicine & Surgery and Pathobiology and Integrative Biomedical Sciences, College of Veterinary Medicine, University of Missouri, Columbia, Missouri, United States; 3 Mason Eye Institute, School of Medicine, University of Missouri, Columbia, Missouri, United States

**Keywords:** cornea wound healing, mitochondria, mtDNA damage, mitochondrial transcription factor A, oxidative stress, fibroblast to myofibroblast transdifferentiation

## Abstract

**Purpose:**

Mitochondria regulate cellular activity in a tissue-selective manner. The role of mitochondria in corneal fibrosis is elusive. This study investigated changes in mitochondrial DNA (mtDNA) and mitochondrial transcription factor A (TFAM) in human corneal stromal fibroblasts (CSFs) and corneal myofibroblast (CMFs) and effects on corneal fibrosis in vitro and ex vivo.

**Methods:**

Healthy donor human corneas were used to generate CSFs and ex vivo culture*.* CMF formation was induced by transforming growth factor beta-1 (TGFβ1) in vitro and human cornea by nitrogen mustard (NM) ex vivo. mtTFA/TFAM CRISPR/Cas9 KO plasmid, Lipofectamine CRISPRMAX, and TrueCut Cas9 Protein v2 were used for gene editing. Long-range PCR and quantitative reverse-transcription PCR (qRT-PCR) measured mtDNA transcription, mtDNA quantity, and ratios of mtDNA to nuclear DNA (nDNA). Immunofluorescence and immunoblotting quantified protein expression. The MitoSOX assay was used to analyze mitochondrial reactive oxygen species (mtROS).

**Results:**

Human CMFs showed significantly reduced mtDNA copies (*P* < 0.01) and mtDNA-to-nDNA ratios (*P* < 0.05) compared to CSFs. Significant time-dependent increases in mRNA levels of α-smooth muscle actin (αSMA) and nDNA-transcribed genes and decreases in TFAM and mtDNA-transcribed genes were noted during CSF transdifferentiation to CMFs (*P* < 0.05, *P* < 0.001, or *P* < 0.0001). Correspondingly, time-dependent decreases in TFAM and increases in Rieske iron–sulfur (Fe-S) and αSMA protein (*P* < 0.0001) and mtROS and ROS levels (*P* < 0.0001) were observed. TFAM silencing arrested fibrotic events and exhibited reduced αSMA and enhanced mtDNA (*P* < 0.001). The NM-induced fibrotic human cornea showed decreased TFAM and increased αSMA compared to naïve corneas (*P* < 0.01).

**Conclusions:**

We observed that mtDNA plays an important role in corneal fibroblast transdifferentiation to myofibroblast and that TFAM has the potential to modulate this process in an injured cornea. Additional studies are warranted.

Corneal myofibroblasts (CMFs) are the major facilitators of corneal tissue repair after injury, trauma, or infection.^1^^,^^2^ CMFs are generated from keratocytes/fibroblasts (corneal stromal fibroblasts [CSFs]), the resident cells of the corneal stroma, after injury under influence of transforming growth factor beta-1 (TGFβ1) and other growth factors in the cornea.^3^ Although formation of CMFs is paramount for corneal wound healing, their excessive formation and persistence in stroma after wound closure result in scarring and vision impairment.^4^ Unlike CSFs, CMFs are characterized by a significant increase in α-smooth muscle actin (αSMA) levels, the ability to enhance extracellular matrix (ECM) protein production, low optical density, and high metabolic activity.^5^^,^^6^ These CMF properties prompted us to postulate that mitochondria in CSFs undergo significant modifications during transdifferentiation to CMFs to meet increased energy demands for sustaining newly attained contractile phenotype and function by increasing aerobic glycolysis and oxidative phosphorylation during corneal wound healing. Also, mitochondrial biogenesis and mitophagy play a key role in proper CMF functioning.

A mitochondrion is a sequestered organelle within a cell known as the powerhouse of the cell for its adenosine triphosphate (ATP) production function.^7^ However, in addition to ATP production, mitochondria regulate many essential signaling molecules, including calcium (Ca^2+^), reactive oxygen species (ROS), intrinsic/extrinsic cell death transcription factors, and inflammatory cytokine transduction.^8^^–^^12^ Furthermore, mitochondria have a highly dynamic nature that allows them to change in size, shape, and position to meet the metabolic needs of the cell.^13^ Thus, mitochondria contribute to tissue and event-specific changes during wound healing or repair processes.^14^^–^^18^ To adapt to cell stress and maintain a self-sustaining function, mitochondria use their own DNA (mtDNA). The mtDNA is double stranded and circular and encodes polypeptides, transfer RNAs (tRNAs), and ribosomal RNAs (rRNAs) that regulate mitochondrial and intracellular protein production and post-translational modifications.^19^ The loss of mtDNA is a frequent cause of mitochondrial dysfunction.^20^^,^^21^

The role of mtDNA in ocular diseases was first noted when mutations in mtDNA were linked to ocular degenerative diseases including Kearns–Sayre syndrome (KSS), Leber hereditary optic neuropathy, and corneal dystrophies including keratoconus.^22^^–^^24^ Recent studies from our laboratory have indicated the involvement of mitochondrial signaling in CMF-induced production of ECM proteins and ferroptotic cell death in rabbit and human corneas after mustard gas injury.^25^^–^^28^ Despite their multifaceted roles during cell stress, mitochondria have a limited repair capacity, making mtDNA highly susceptible to damage.^29^^,^^30^ Proper mitochondrial function relies on crucial protective factors such as mitochondrial transcription factor A (TFAM), which is encoded in the nucleus but is transported into the mitochondria to regulate and stabilize mtDNA.^31^^,^^32^ The current literature lacks information about the status of mtDNA and TFAM in CSFs and CMFs and their role in corneal wound healing. This study investigated changes in mtDNA and TFAM occurring during CMF formation from CSFs, as well as their role in myofibroblast and fibrosis development using clustered regularly interspaced short palindromic repeats (CRISPR)/CRISPR-associated protein 9 (Cas9) gene editing technology, and we developed human cornea fibrosis in vitro and ex vivo organ culture models.

## Materials and Methods

### In Vitro and Ex Vivo Human Cornea Cultures

Healthy human cadaver corneas from 40- to 70-year-old donors were purchased from Saving Sight (Kansas City, MO, USA) and were used for ex vivo experiments and to generate primary CSFs for in vitro experiments using our published protocol.^33^ In brief, primary CSFs were generated by gently removing the epithelium and endothelium from the cornea. The stromal tissues were then cut into small pieces, washed, and placed on a 60-mm culture dish containing 1 mL CSF medium supplemented with 10% fetal bovine serum, and incubated at 37°C in a humidified CO_2_ incubator. The medium included Gibco Minimum Essential Media (61100061; Thermo Fisher Scientific, Waltham, MA, USA), Gibco MEM Amino Acids Solution (11130051; Thermo Fisher Scientific), Gibco MEM Vitamin Solution (11120052; Thermo Fisher Scientific), Gibco Sodium Pyruvate (11360070; Thermo Fisher Scientific), and Gibco Antibiotic-Antimycotic solution (15240062; Thermo Fisher Scientific). After CSFs sprouted from stromal explants and reached 70% confluence, cells were harvested and used for the experiments. No CSFs after passage three were used in the study. CMFs were generated by growing CSFs in vitro in TGFβ1 (5 ng/mL) or ex vivo using nitrogen mustard (NM; 100 ng/mL) for 72 hours at 37°C in a humidified 5% CO_2_ incubator. Cultures were fed fresh CSF medium and TGFβ1 or NM every 24 hours.^34^ The human cornea ex vivo organ culture was set up by placing the donor cornea onto a custom corneal conformer matching normal corneal curvature without damaging the corneal endothelium into a six-well tissue culture dish. Medium was added to a level such that the corneosclera of the tissue remained continually bathed in the medium but the axial cornea remained exposed to the air. Cultures were kept at 37°C with a 5% CO_2_ humidified incubator for 7 days. The medium was changed every 24 hours, and the axial cornea received one drop of medium three times daily (6 AM, 2 PM, and 10 PM) to prevent corneal desiccation. Corneal tissues were exposed to NM (100 ng/mL) or vehicle (medium) for 72 hours. The NM solution was prepared by reconstituting mechlorethamine hydrochloride (122564; Sigma-Aldrich, St. Louis, MO, USA) in culture media.

### CRISPR/Cas9 Gene Transfer

The mitochondrial transcription factor A (mtTFA) CRISPR/Cas9 Double Nickase plasmid (sc-401208-NIC; Santa Cruz Biotechnology, Dallas, TX, USA) was introduced into human CSFs via Lipofectamine CRISPRMAX Cas9 Transfection Reagent (CMAX00001; Thermo Fisher Scientific) following the manufacturer's instructions. The TrueCut Cas9 Protein v2 (A36498; Thermo Fisher Scientific) was used for its superior cleavage efficiency and effective gene editing. In brief, CSFs were starved overnight, followed by treatment with a CRISPRMAX Cas9/TFAM/green fluorescent protein (GFP) complex for 48 hours and puromycin selection (1 µg/mL) following the manufacturer's instructions. Transfection efficiency and complete TFAM silencing in cultures were verified by the immunofluorescence and PCR techniques (Supplementary Fig. S1). The transfection efficiency was 45% ± 5% based on the ratio of GFP^+^ and 4′,6-diamidino-2-phenylindole (DAPI)^+^ cells to GFP^–^DAPI^+^ cells in a 100× magnification field.

### Mitochondrial ROS Assay

To measure mitochondrial reactive oxygen species (mtROS) in human CSFs (hCSFs) in vitro, hCSFs were plated onto a clear 96-well plates in ±TGFβ1 (5 ng/mL) in serum-free medium for 24 hours, 48 hours, and 72 hours. The mtROS levels were measured using Invitrogen MitoSOX Mitochondrial Superoxide Indicators (M36008; Thermo Fisher Scientific) following the manufacturer's instructions. After incubation, 100 mL of working solution was added to all wells. The plates were incubated in the dark for 30 minutes, and stop solution was then added. Absorbance at 490 nm was measured using an Agilent BioTek Synergy H1 multimode reader (BTH1M2FG; Agilent Technologies, Santa Clara, CA, USA). An equal number of cells (3 × 10^3^ per well) were plated during the MitoSOX assay based on established methods.^35^

### Immunofluorescence

Cultures were fixed at 24 hours, 48 hours, and 72 hours with 4% paraformaldehyde (PFA) for 30 minutes, washed with filtered PBS three times, and then blocked with 5% BSA for 30 minutes at room temperature. Thereafter, cells received three more washes with PBS and the primary TFAM antibody at 1:200 dilution (8076T; Cell Signaling Technology, Danvers, MA, USA) or αSMA at 1:500 (GA611; Agilent Technologies) overnight at 4°C. Cultures were washed three times after the primary antibody was removed, followed by Goat anti-Rabbit IgG (H+L) Cross-Adsorbed Secondary Antibody, Alexa Fluor 594 (1:500 dilution; A-11012; Thermo Fisher Scientific) or Goat anti-Mouse IgG (H+L) Cross-Adsorbed Secondary Antibody, Alexa Fluor 488 (1:500 dilution; A-11001; Thermo Fisher Scientific) for 4 hours at room temperature. Rabbit (DA1E) mAb IgG XP Isotype Control (3900S; Cell Signaling Technology) was used for TFAM and Invitrogen Mouse IgG2a, kappa Isotype Control (14-4724-82; Thermo Fisher Scientific) was used for αSMA as isotype controls.

### RNA Extraction, cDNA Synthesis, and Quantitative Reverse-Transcription PCR

The relative change in mRNA expression was measured using quantitative reverse-transcription PCR (qRT-PCR). Total RNA was extracted from CSFs in Buffer RLT (79216; QIAGEN, Hilden, Germany) using the RNeasy Mini Kit (74104; QIAGEN) following the manufacturer's instructions. Extracted total RNA was quantified and converted into cDNA using GoTaq Flexi DNA Polymerase (M8291; Promega, Madison, WI, USA). The expression levels of the mtDNA-encoded genes were determined using NADH-ubiquinone oxidoreductase chain 6 (ND6), ND1, and cytochrome c oxidase subunit I (COX1). The expression levels of nuclear-encoded genes were determined using DNA polymerase subunit gamma (POLG), TFAM, and mtDNA-directed RNA polymerase (POLRMT) were quantified with qRT-PCR using the StepOnePlus Real-Time PCR System (Applied Biosystems, Carlsbad, CA, USA). The primer sequences for qRT-PCR of the tested genes are listed in the Table. Glyceraldehyde 3-phosphate dehydrogenase (GAPDH) was used as a housekeeping gene, and the relative fold change was calculated using our established methods.^36^ The qRT-PCR was run in triplicate each time, and the average of three independent experiments has been provided in Figures 2 and 3.

**Table.** Accession Numbers and Sequences Used for qRT-PCR

Sequence No.	Gene Name	Accession No.	Forward Sequence (5′–3′)	Reverse Sequence (3′–5′)
1	*GAPDH*	NM_002046.3	GACCTGACTGACTACCTCAT	ATGTCACGCACGATTTCC
2	*αSMA*	NM_001141945	AAGATCCTGACTGAGCGT	CAAAGTCCAGAGCGACATAG
3	*TFAM*	NM_001270782	CTCAGAACCCAGATGCAA	TCAGGAAGTTCCCTCCAA
4	*COX1*	NC_001224.1	TTAGCTGACTCGCCACACTC	GGCCACCTACGGTGAAAAGA
5	*ND1*	NC_012920.1	ACTACGCAAAGGTCCCAACG	GGCGGGTTTTAGGGGTTCTT
6	*ND6*	NC_024511.2	TCAACGCCCATAATCATACAAA	GATGGCTATTGAGGAGTATCCTGAG
7	*PI3K*	XM_017342488.1	GCAACCCAGAACTGATAGTG	GGAAGGTTGCAGTCCATAAG
8	*AKT*	NM_001382431.1	GTCTCTGCCTTGGACTATCT	GGCCATCTTTATCCAGCATC
9	*POLRMT*	NM_005035	GGGACCATCGAAAGGTGTCT	CTTCAGAACAGTGGCCCGAT
10	*mtDNA*	NC_012920.1	CCTGTACGAAAGGACAAGAG	GGAAGCGGATGAGTAAGAAG

### DNA Extraction, Long-Range PCR, and mtDNA Quantification

Cells were scraped from plates and collected in PBS, and DNA was extracted using DNeasy Blood and Tissue Kits for DNA Isolation (69504; QIAGEN). Long-range polymerase chain reaction (LX-PCR) was performed to detect mtDNA damage using a Phusion High-Fidelity PCR Kit (F553L; Thermo Fisher Scientific) following the manufacturer's protocol. The forward primer (5′–CAG TGC AGT GCT TGA TAA CAG G–3′) and reverse primer (5′–GTA GTG CGC GTT TGA TTT CC–3′) are provided in the manufacturer's kit. Twenty-microliter reactions contained 4 µL of 5× Phusion HF Buffer, 0.4 µL of 10-mM deoxyribonucleotide triphosphate (dNTP), 1 µL of forward primer, 1 µL of reverse primer, 2 µL of template DNA, 0.6 µL of dimethyl sulfoxide (DMSO), and 0.2 µL of Phusion DNA Polymerase. Following the manufacturer's guidelines, the reaction mixture was cycled using a three-step protocol. Initial denaturation occurred at 98°C for 3 minutes followed by 25 cycles of 10 seconds at 98°C, 30 seconds at 60°C (melting temperature of the primer for less than 20 cycle threshold), 11 minutes at 68°C, and a final elongation for 10 minutes at 72°C. LX-PCR products were separated on a 0.5% agarose gel by electrophoresis stained with ethidium bromide. LX-PCR product was extracted from gel using a QIAquick Gel Extraction Kit (28704; QIAGEN) following the manufacturer's instructions and confirmed to be derived from mtDNA via restriction enzyme analysis. In brief, 500 ng of LX-PCR amplicon was incubated at 37°C overnight with FastDigest BgIII (FD0083; Thermo Fisher Scientific) endonuclease and provided buffer in a final volume of 50 µL. The DNA fragments were separated on a 1.2% agarose gel, and the gel was imaged using an iBright CL1500 Imaging System (Thermo Fisher Scientific). The NovaQUANT Human Mitochondrial to Nuclear DNA Ratio Kit (72620Kit; Sigma-Aldrich) and a mitochondrial DNA copy kit (Detroit R&D, Detroit, MI, USA) were used to quantify the ratio of mtDNA to nDNA and mtDNA, respectively.

### Protein Extraction and Western Blotting

Total protein was prepared from each cell lysate collected in radioimmunoprecipitation assay (RIPA) Lysis and Extraction Buffer (89900; Thermo Fisher Scientific) and quantified using Pierce Bradford Plus Protein Assay Reagent (23238; Thermo Fisher Scientific). Protein samples were heated at 90°C for 5 minutes, separated on a NuPAGE Bis-Tris Mini Protein Gels, 4-12% (NP0321PK2; Thermo Fisher Scientific), transferred to a nitrocellulose membrane (LC2006; Thermo Fisher Scientific), washed with Tris-buffered saline (TBS), then blocked for 1 hour with 5% fat-free milk at room temperature. After blocking, the membrane was washed three times with TBS with Tween 20 (TBST) for 10 minutes; incubated with primary antibody at a 1:100 dilution of TFAM (8076T; Cell Signaling Technology), αSMA (M0851; Agilent), or Rieske Fe-S (sc-271609; Santa Cruz Biotechnology); and incubated overnight at 4°C. Membranes were then washed three times with TBST for 10 minutes each. Horseradish peroxidase–conjugated anti-mouse antibody (1:2000) was added and membrane was incubated for 4 hours at room temperature. The immunoblots were incubated for 5 minutes with SuperSignal West Dura Extended Duration Substrate (37071; Thermo Fisher Scientific) and then imaged and analyzed using an iBright CL1500 Imaging System (Thermo Fisher Scientific).

### Statistical Analysis

Prism 10.1 (GraphPad, Boston, MA, USA) software was used for statistical analysis. Each experiment was conducted at least three times, and each test sample was run in triplicate. Student’s *t*-test and one- or two-way analysis of variance (ANOVA) with Bonferroni post hoc tests were used, as needed, during statistical analysis. The result values are expressed as mean ± SEM. A value of *P* < 0.05 was considered statistically significant.

## Results

### Quantification of mtDNA in Human Corneal Fibroblasts and Myofibroblasts

**Figure 1.**
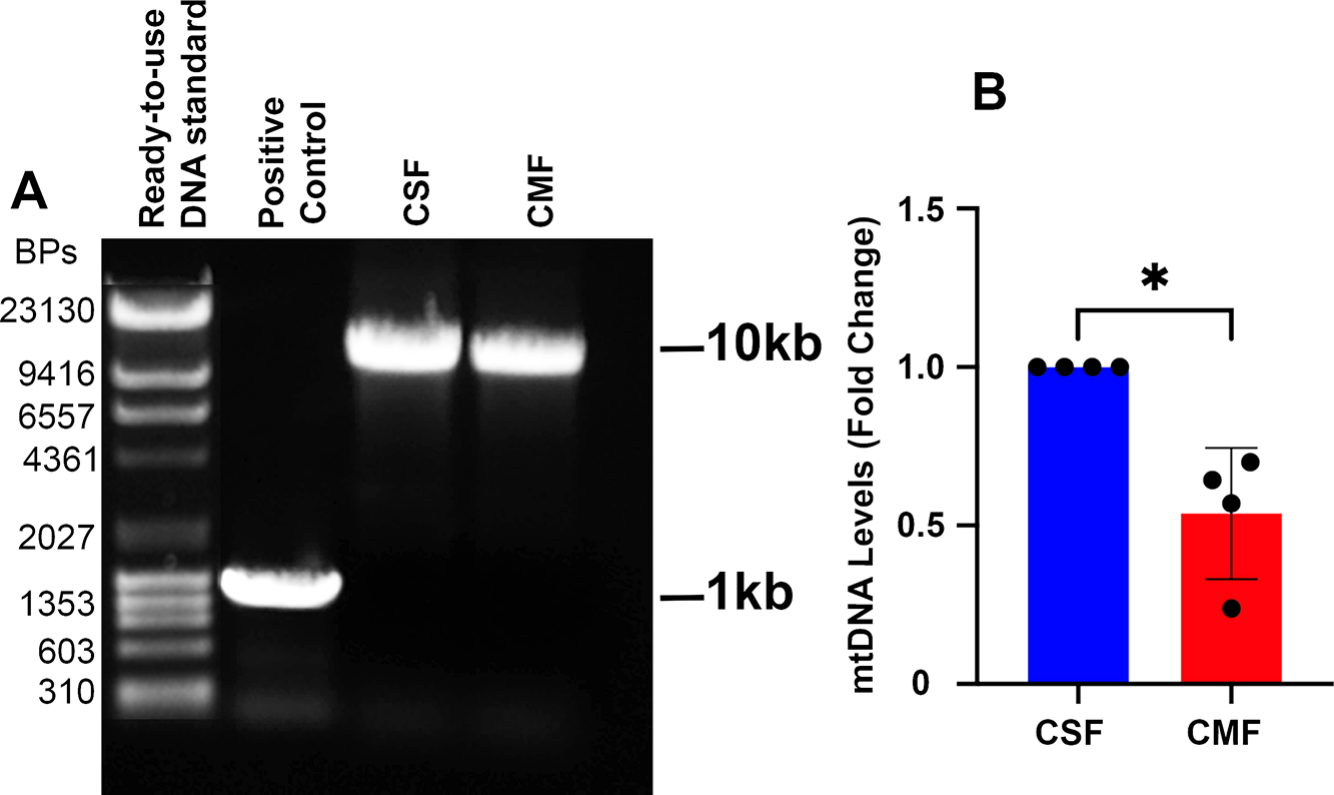
**Corneal myofibroblasts have significantly reduced mtDNA compared to corneal fibroblasts.** (**A**) LX-PCR detected the amount of mtDNA present in human CSFs and CMFs. (**B**) Densitometry analysis of mtDNA bands showed that CMFs had significantly decreased mtDNA in comparison to CSFs. Data shown were collected from four sets of primary cultured CSFs and CMFs, with four separate cell cultures per group and samples tested in triplicate. *Error bars* depict standard deviations. One primary culture set was derived from one donor human cornea. Ready-to-use DNA standards and positive controls were provided in the Phusion High-Fidelity PCR Kit. *P < 0.05.

**Figure 2.**
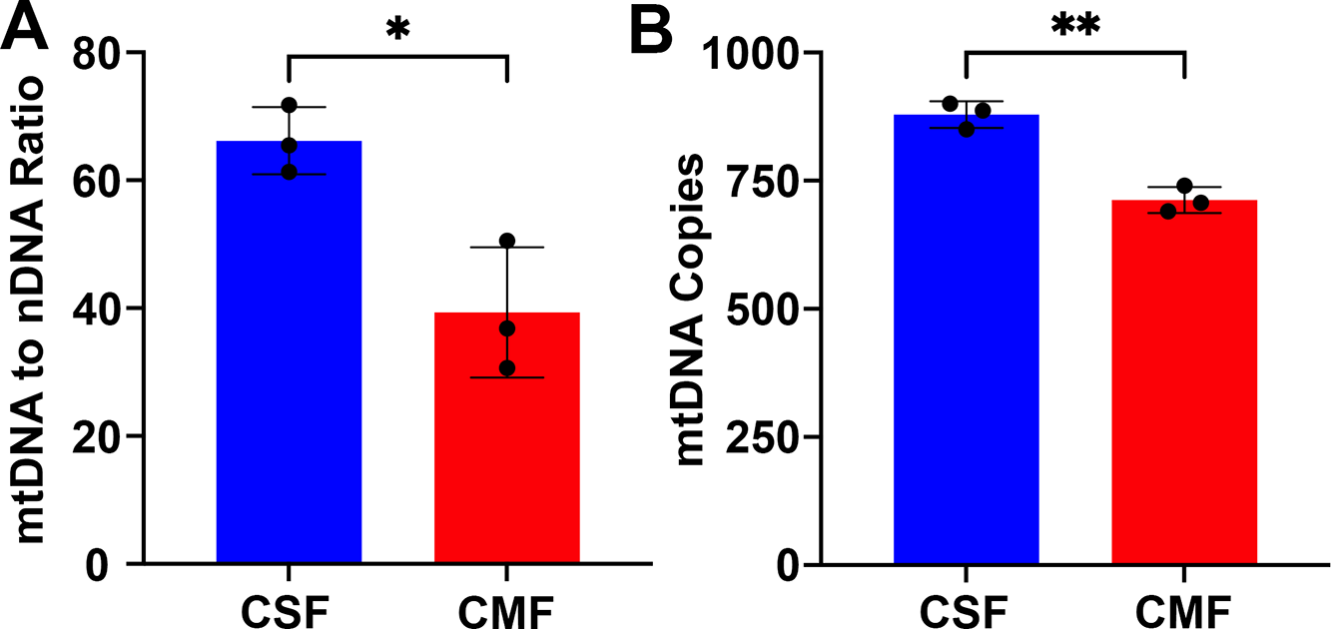
**CMFs have decreased mtDNA-to-nDNA ratios and total mtDNA copies in vitro.** (**A**, **B**) The mtDNA was quantified for CSFs and CMFs using the NovaQUANT Human Mitochondrial to Nuclear DNA Ratio Kit (**A**) and the Detroit R&D mitochondrial DNA copy kit (**B**). Human CMFs have significantly decreased mtDNA-to-nDNA ratios (**A**) and mtDNA copies (**B**). Data shown are from three separate cell cultures (isolated from three separate donor corneas) per group with samples tested in triplicate. *Error bars* depict standard deviations. *P < 0.05, **P < 0.01.

Figures 1 and 2 show the quantification of mtDNA and ratios of mtDNA to nDNA in human CSFs and CMFs in vitro, respectively. The CSFs transdifferentiated into CMFs in the presence of TGFβ1 at 72 hours under serum-free conditions. A significantly reduced mtDNA expression was observed in CMFs compared to the CSFs, as evident from the amplicon size (10 kb) determined by gel electrophoresis (Fig. 1A) and quantification analysis (Fig. 1B) (*P* < 0.05). The mtDNA amplification was confirmed by restriction enzyme mapping, which revealed correctly sized DNA fragments based on gel electrophoresis (Supplementary Fig. S2). Additionally, the CMFs showed a significantly decreased ratio of mtDNA to nDNA (Fig. 2A) (*P* < 0.05) and mtDNA copies (Fig. 2B) (*P* < 0.01) compared to the CSFs. This analysis revealed that the decrease in mtDNA transcription and quantity in CMFs is due to reduced mtDNA copies. The ratio of mtDNA to nDNA was identified as reported previously.^37^

### Time-Dependent Alterations in Transcript Expression of αSMA, TFAM, mtDNA-Transcribed Genes and nDNA-Transcribed Genes in CSFs in ±TGFβ1

**Figure 3.**
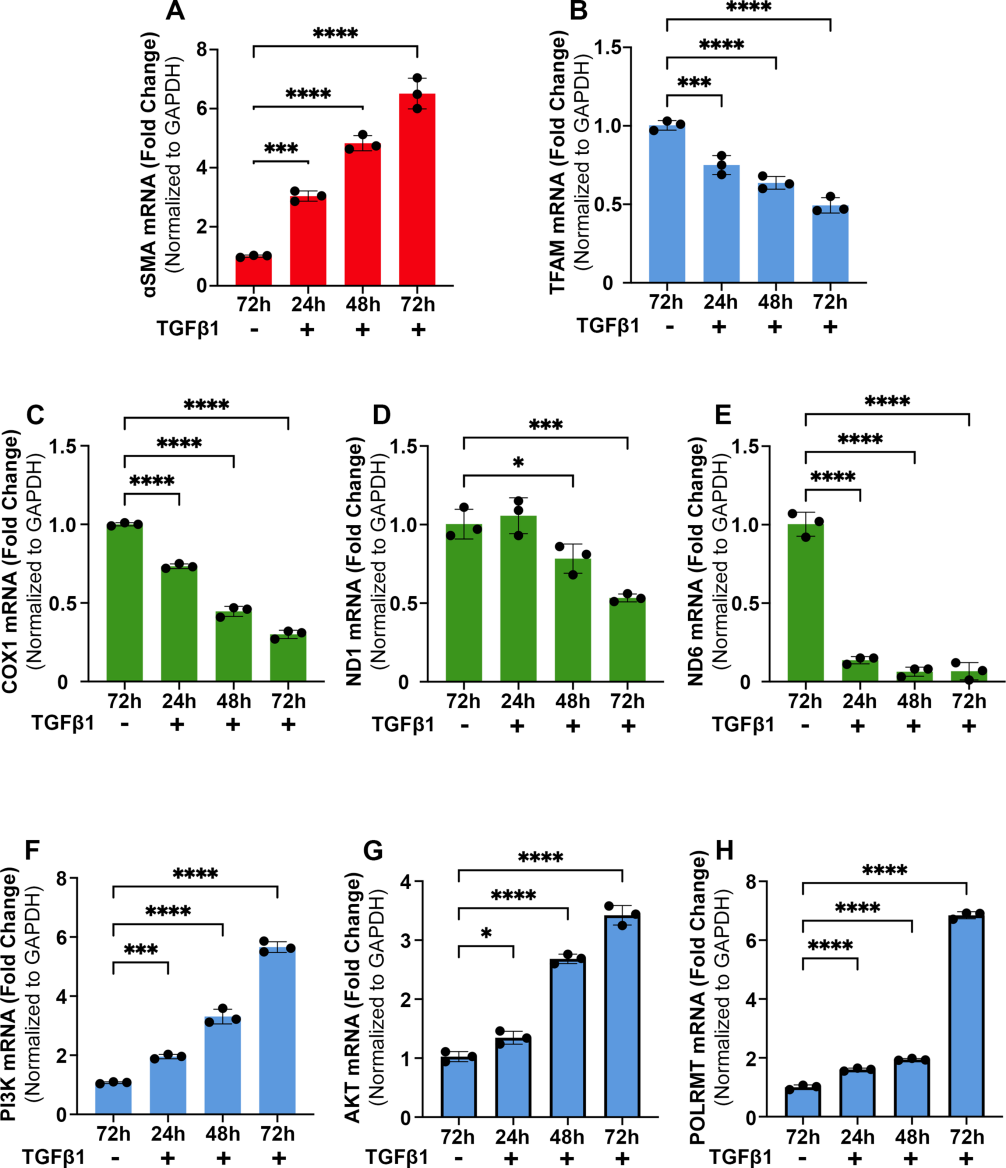
**Time-dependent alterations in transcript expression of αSMA, TFAM, mtDNA-transcribed genes, and nDNA-transcribed genes in CSFs ±TGFβ1 in vitro.** (**A**, **B**) Human CSFs cultured with TGFβ1 under serum-free conditions had a significant increase in mRNA levels of αSMA (**A**) at 24 hours (*P* < 0.001), 48 hours (*P* < 0.0001), and 72 hours (*P* < 0.0001) and a significant decrease in mRNA levels of TFAM (**B**) at 24 hours (*P* < 0.001), 48 hours (*P* < 0.0001), and 72 hours (*P* < 0.0001) compared to CSFs cultured without TGFβ1. (**C**–**E**) Transcription of the mtDNA-transcribed genes ND1 (**C**), ND6 (**D**), and COX1 (**E**) was significantly decreased at 24 hours (*P* < 0.0001), 48 hours (*P* < 0.0001), and 72 hours (*P* < 0.0001) in CSFs cultured with TGFβ1 compared to CSFs without TGFβ1. (**F**–**H**) Conversely, the transcription of nuclear proteins related to mitochondrial function or oxidative stress, PI3K (**F**), AKT (**G**), and POLRMT (**H**), was significantly increased at 24 hours (*P* < 0.05, *P* < 0.001, or *P* < 0.0001), 48 hours (*P* < 0.0001), and 72 hours (*P* < 0.0001) in CSFs cultured with TGFβ1 compared to CSFs without TGFβ1. The CSFs cultured in the absence of TGFβ1 had no significant alterations in αSMA (**A**), TFAM (**B**), mtDNA-transcribed genes (**C**–**E**), or nDNA-transcribed genes (**F**–**H**) at 24 hours, 48 hours, and 72 hours (data not shown). To simplify data presentation in graphs, representative 72-hour data for CSFs cultured without TGFβ1 are shown in panels **A** to **H**. Data shown are from three separate cell culture experiments (isolated from three separate donor corneas) per group, with each sample run in triplicate. *Error bars* depict standard deviations. *P < 0.05, ***P < 0.001, ****P < 0.0001.

Figure 3 shows changes in mRNA expression of αSMA (Fig. 3A), TFAM (Fig. 3B), mtDNA, mtDNA-transcribed genes (Figs. 3C–E), and nDNA-transcribed genes (Figs. 3F–H) in CSFs at 24 hours, 48 hours, and 72 hours in the presence or absence of TGFβ1. CSFs cultured in the presence of TGFβ1 showed a progressive increase in αSMA transcript levels at 24 hours (*P* < 0.001), 48 hours (*P* < 0.0001), and 72 hours (*P* < 0.0001) (Fig. 3A) compared to CSFs cultured in the absence of TGFβ1. In contrast, CSFs grown in the presence of TGFβ1 had significantly decreased TFAM transcript levels under similar conditions compared to the CSFs grown in the absence of TGFβ1 at 24 hours (*P* < 0.001), 48 hours (*P* < 0.0001), and 72 hours (*P* < 0.0001) (Fig. 3B). Furthermore, CSFs cultured in the absence of TGFβ1 had no significant alterations in αSMA (Fig. 3A), TFAM (Fig. 3B), mtDNA-transcribed genes (Figs. 3C–E), or nDNA-transcribed genes (Figs. 3F–H) at 24 hours, 48 hours, and 72 hours (data not shown). Thus, to simplify data presentation, only 72-hour data for CSFs without TGFβ1 are shown in Figures 3A to 3H.

The changes in transcript expression of mtDNA-transcribed genes tested include ND1, ND6, and COX1. TGFβ1 treatment of CSFs caused a significant decrease in COX1 (Fig. 3C) at 24 hours (*P* < 0.0001), 48 hours (*P* < 0.0001), and 72 hours (*P* < 0.0001); ND1 (Fig. 3D) at 48 hours (*P* < 0.05) and 72 hours (*P* < 0.001); and ND6 (Fig. 3E) at 24 hours (*P* < 0.0001), 48 hours (*P* < 0.0001), and 72 hours (*P* < 0.0001).

The changes in transcript expression of nDNA-transcribed genes, phosphoinositide 3-kinase (PI3K), protein kinase B (AKT), and POLRMT were measured. TGFβ1 treatment of CSFs caused a significant increase in PI3K (Fig. 3F) at 24 hours (*P* < 0.001), 48 hours (*P* < 0.0001), and 72 hours (*P* < 0.0001); AKT (Fig. 3G) at 24 hours (*P* < 0.05), 48 hours (*P* < 0.0001), and 72 hours (*P* < 0.0001); and POLRMT (Fig. 3H) at 24 hours (*P* < 0.0001), 48 hours (*P* < 0.0001), and 72 hours (*P* < 0.0001). These data indicate a relationship among mtDNA, mtDNA-to-nDNA ratio, and TFAM during human CSF transdifferentiation into CMFs in vitro.

### Time-Dependent Changes in αSMA, TFAM, and Mitochondrial Protein Expression in CSFs in ±TGFβ1

**Figure 4.**
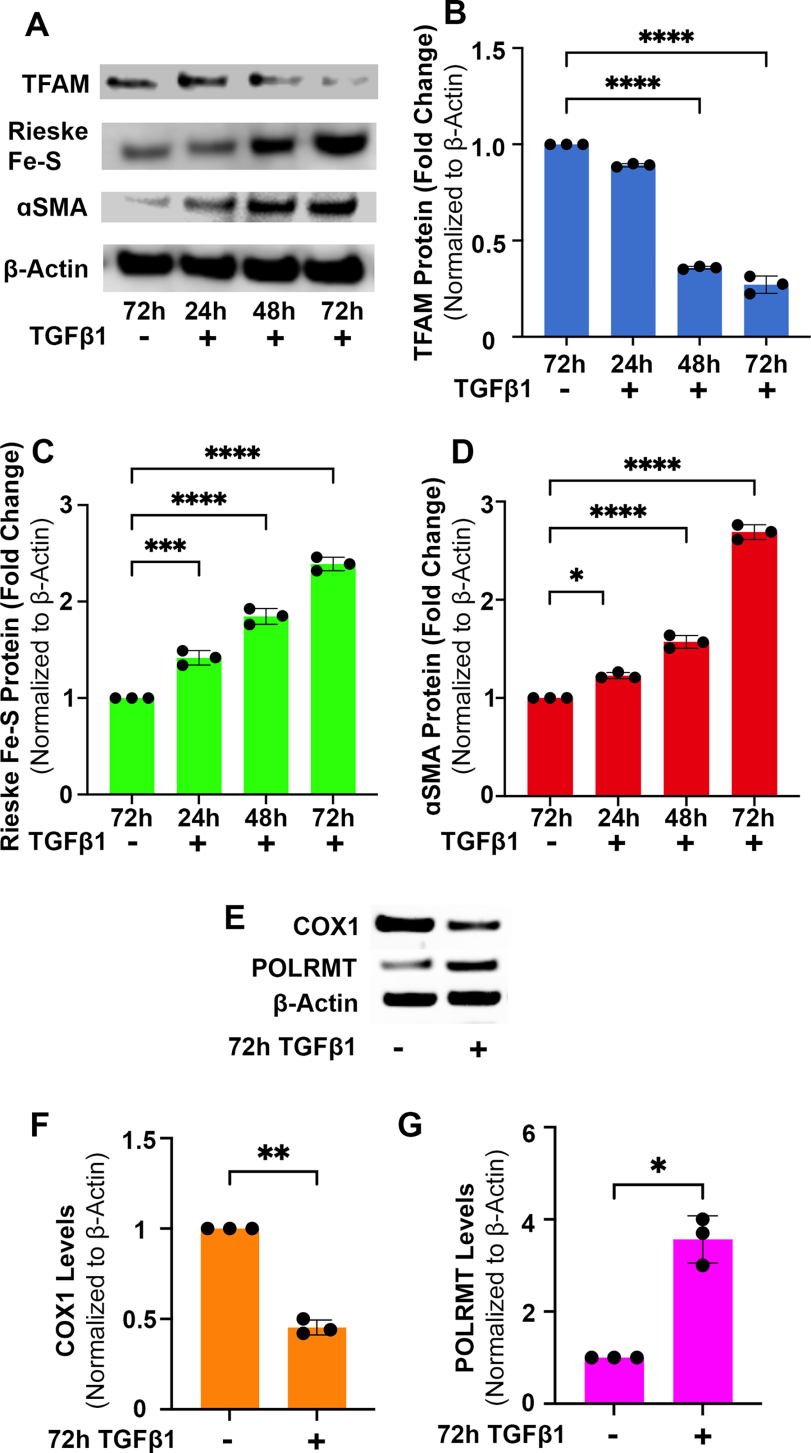
**Time-dependent changes in** **αSMA, TFAM, and mitochondrial proteins levels in CSFs in** **±TGFβ1** **in vitro**. (**B**, **C**) Human CSFs cultured with TGFβ1 in a serum-free environment showed a significant decrease in protein levels of TFAM (**B**) at 24 hours (*P* < 0.05), 48 hours (*P* < 0.0001), and 72 hours (*P* < 0.0001) and a significant increase in protein levels of Rieske Fe-S (**C**) at 24 hours (*P* < 0.001), 48 hours (*P* < 0.0001), and 72 hours (*P* < 0.0001). (**D**) Rieske Fe-S is a membrane protein subunit generated in the mitochondria; shown is the decrease in TFAM and increase in Rieske correlated with a time-dependent increase in αSMA. Representative western blots of TFAM, Rieske Fe-S, αSMA, and β-Action are shown in panel (**A**). Further, western blotting analysis detected a significant decrease in protein expression of the mtDNA-transcribed gene COX1 (**F**) and a significant increase in the nDNA-transcribed gene POLRMT (**G**) after 72 hours of TGFβ1 (*P* < 0.01 and *P* < 0.05, respectively). Representative western blots of COX1, POLRMT, and β-Actin are shown in panel (**E**). Findings confirmed that the loss of mtDNA corresponds with a decrease in mtDNA transcription and translation. Quantification is shown in the graphics. Data shown are from three separate cell cultures (isolated from three separate donor corneas). *Error bars* depict standard deviations. *P < 0.05, **P < 0.01, ***P < 0.001, ****P < 0.0001.

Figure 4 shows western blot images of TFAM, Rieske Fe-S, and αSMA (Fig. 4A) and quantification of detected bands (Figs. 4B–D). The CSFs treated with TGFβ1 showed a progressive significant decrease in TFAM but a significant increase in Rieske Fe-S and αSMA protein expression levels at 24 hours, 48 hours, and 72 hours while transdifferentiating into CMFs (Fig. 4A). Quantification analysis found a significant gradual decrease in TFAM due to TGFβ1 at 48 hours (*P* < 0.0001) and 72 hours (*P* < 0.0001) compared to control CSFs (grown in the absence of TGFβ1). Conversely, progressive significant increases in Rieske Fe-S (Fig. 4C) and αSMA (Fig. 4D) protein expression at 24 hours (*P* < 0.001), 48 hours (*P* < 0.0001), and 72 hours (*P* < 0.0001) were observed in CSFs with TGFβ1 exposure compared to control CSFs (grown in the absence of TGFβ1). Rieske Fe-S is an exclusive mitochondrial membrane protein and was used to analyze changes in mitochondrial quantity.^38^ This analysis indicated that the number of mitochondria increased but TFAM, a mtDNA regulatory factor, decreased during CSF transdifferentiation into CMFs in vitro.

The western blot analysis of CSFs grown with or without TGFβ1 for 72 hours showed protein expression of the mtDNA-transcribed gene COX1 and nDNA-transcribed gene POLRMT (Fig. 4E). This analysis confirmed the mRNA findings, as COX1 protein was significantly decreased (*P* < 0.01) (Fig. 4F) and POLRMT protein was significantly increased (*P* < 0.05) (Fig. 4G) in CMFs compared to CSFs. These data demonstrate that the loss of mtDNA results in decreased mtDNA protein production.

### Correlation Between mtROS and TFAM Protein Expression in CSFs in ±TGFβ

**Figure 5.**
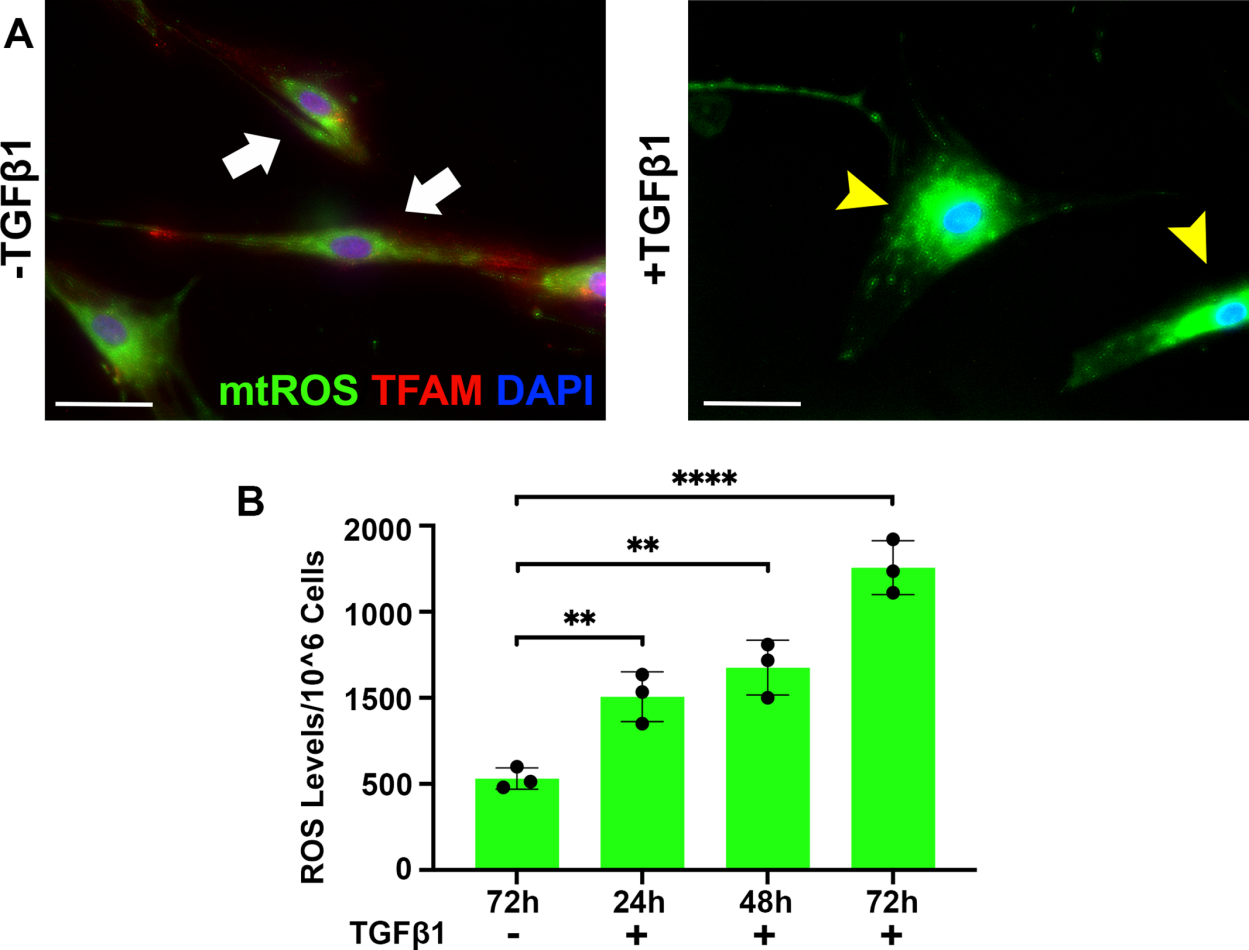
**Correlation between mtROS and TFAM expression in CSFs in ±TGFβ in vitro.** (**A**) The CSFs cultured with or without TGFβ1 were stained for mtROS production with MitoSOX and TFAM. CSFs grown with TGFβ1 had significantly increased MitoSOX green stain (*yellow arrowhead*) compared to CSFs grown without TGFβ1 (*white arrow*). (**B**) MitoSOX analysis of CSFs cultured with TGFβ1 for 72 hours showed a significant time-dependent increase in mtROS production at 24 hours, 48 hours, and 72 hours. Data shown are from three independent experiments with cells isolated from three separate donor corneas, and samples were run in triplicate in every experiment. *Error bars* depict standard deviations. *Scale bar*: 50 µm. *P < 0.05, **P < 0.01.

Figure 5 shows immunofluorescence analyses of TFAM, mtROS, and ROS protein levels in CSFs in ±TGFβ1 at 24 hours, 48 hours, and 72 hours. CSFs grown in TGFβ1 for 72 hours that transformed into CMFs had significantly increased mtROS production and decreased TFAM expression compared to the control CSFs (grown without TGFβ1) in similar experimental conditions. Quantification of immunofluorescence data of CSFs cultured in TGFβ1 revealed a significant decrease in TFAM (Fig. 5B) but an increase in mtROS (Fig. 5C) and ROS (Fig. 5D) levels compared to CSFs grown without TGFβ1. Also, CSFs cultured in TGFβ1 showed a progressive time-dependent increase in ROS levels at 24 hours (*P* < 0.01), 48 hours (*P* < 0.01), and 72 hours (*P* < 0.0001) (Fig. 7D). These data indicate that increased mtROS levels correspond with decreased mtDNA levels in CSFs in vitro.

### TFAM Gene Editing Altered mtDNA Expression in Human CSFs In Vitro

**Figure 6.**
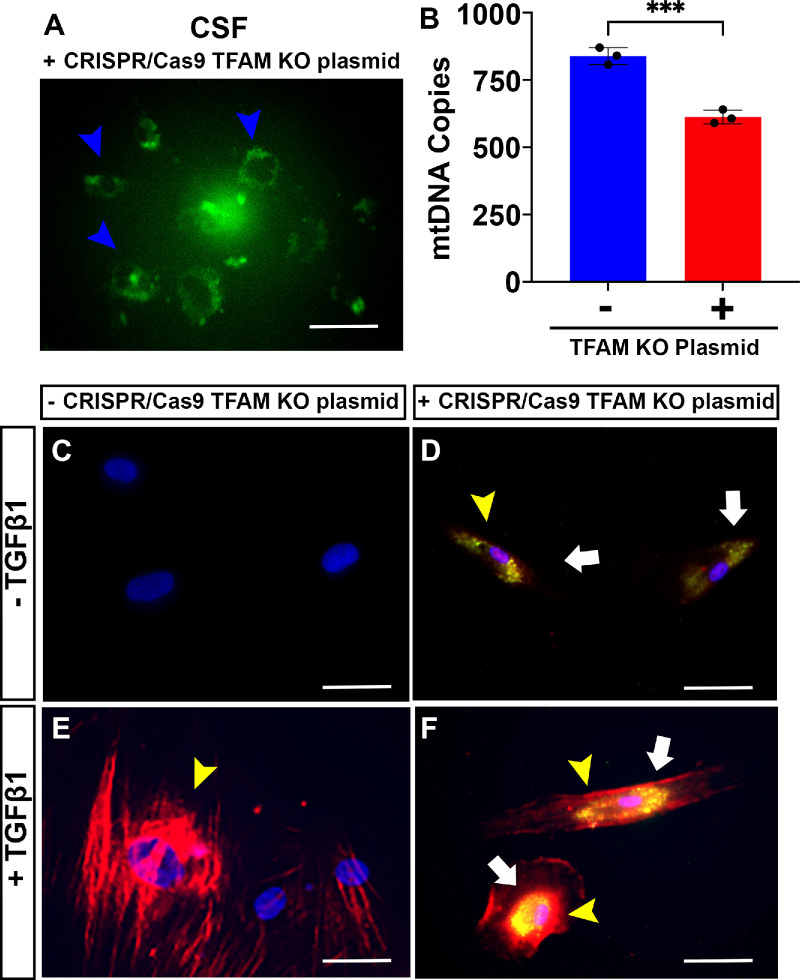
**TFAM gene editing altered mtDNA expression in human CSFs in vitro.** TFAM was silenced in CSFs to ascertain its role in regulating mtDNA. (**A**) Efficient delivery of mtTFA CRISPR/Cas9 KO plasmid into CSFs facilitated gene editing, as evidenced by the detection of GFP tags (*blue arrowheads*). (**B**) Phase contrast imaging identified approximately 90% of human CSF-expressed GFP. TFAM-silenced CSF cultures had significantly reduced mtDNA copies compared to non-silenced normal CSFs. (**E**, **F**) TFAM-silenced CSFs cultured with TGFβ1 (**F**) had significantly decreased αSMA expression compared to non-silenced normal CSFs under similar conditions (**E**). (**C**, **D**) Additionally, TFAM-silenced CSFs cultured without TGFβ1 (**D**) had little increased αSMA expression (*yellow arrowhead*) compared to non-silenced normal CSFs under similar conditions (**C**). These data indicate that silencing of TFAM led to a decrease in mtDNA and an increase in αSMA expression in vitro. Data shown are from three independent experiments, and samples (isolated from three separate donor corneas) were run in triplicate in every experiment. *Error bars* depict standard deviations. TFAM is indicated by a *white arrowhead*. *Scale bar*: 50 µm. ***P < 0.001.

Figure 6 shows successful delivery of mtTFA CRISPR/Cas9 KO plasmid via GFP (Fig. 6A) and the number of mtDNA copies (Fig. 6B) in CSFs. Figures 6C to 6F show the effects of TFAM gene silencing on CSFs grown in the presence or absence of TGFβ1 for 72 hours under a serum-free environment. TFAM was silenced in CSFs to ascertain its role in regulating mtDNA expression. The TFAM-silenced CSFs had significantly reduced mtDNA copies compared to the normal non–TFAM-silenced CSFs (Fig. 6B) (*P* < 0.001). The TFAM-silenced CSFs (Fig. 6F) showed significantly decreased αSMA expression compared to non–TFAM-silenced CSFs (Fig. 6E) (*P* < 0.01) cultured in the presence of TGFβ1 for 72 hours under a serum-free environment. As expected, non–TFAM-silenced normal CSFs grown in the absence of TGFβ1 showed no αSMA expression (Fig. 6C), whereas the TFAM-silenced CSFs revealed a low de novo expression of αSMA (Fig. 6D) under similar experimental conditions. These data indicate that a loss of TFAM leads to a decrease in mtDNA and an increase in αSMA in CSFs in vitro during transdifferentiation.

### Expression of αSMA and TFAM in the Normal and Fibrotic Human Cornea Ex Vivo

**Figure 7.**
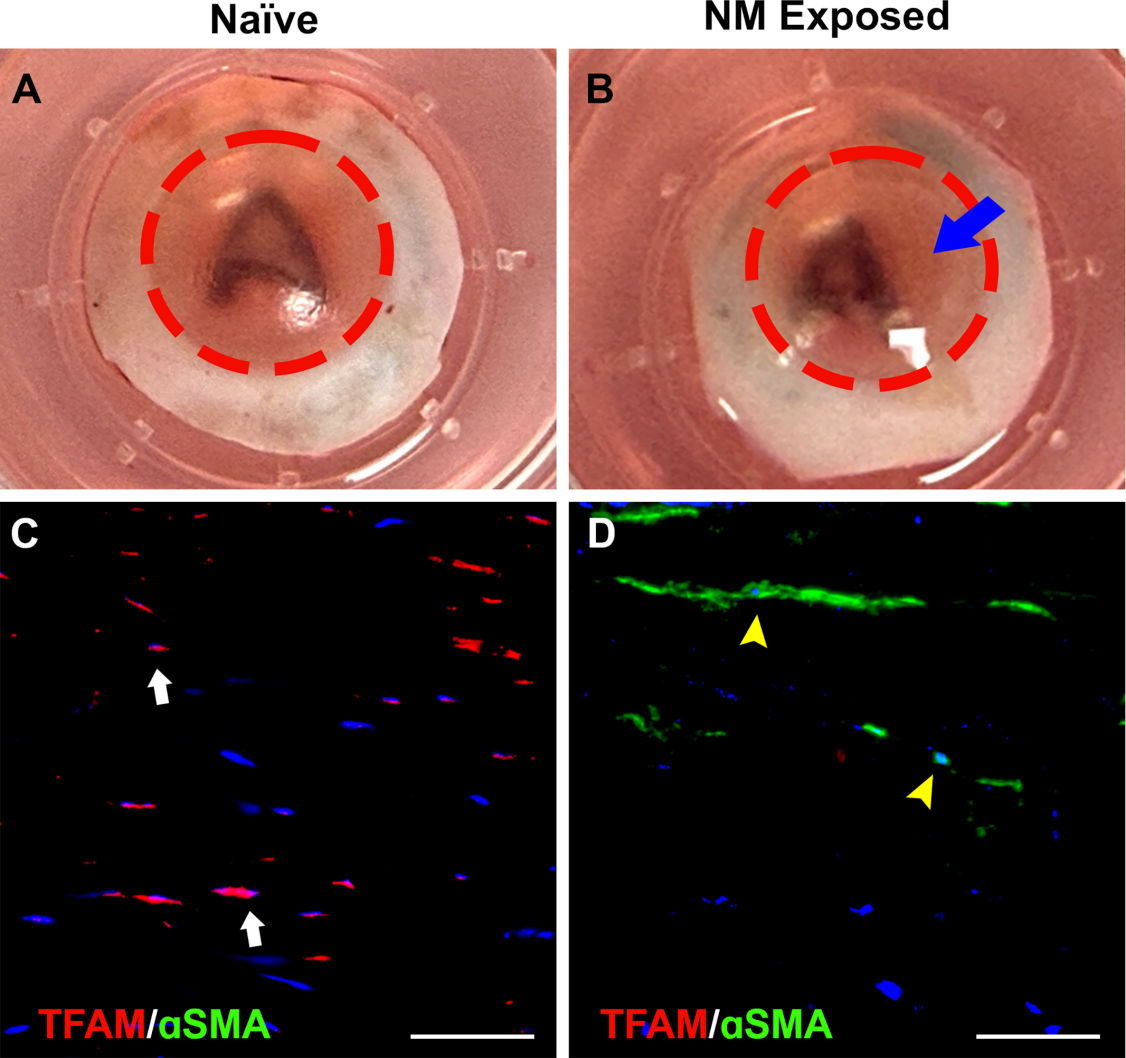
**Expression of αSMA and TFAM in the normal and fibrotic human cornea ex vivo organ culture model.** (**A**–**D**) The opacity levels in normal and fibrotic cornea ex vivo organ culture (**A**, **B**) and αSMA and TFAM levels in stroma of corneal sections (**C**, **D**) at day 7. A blurred letter “A” seen in NM-treated fibrotic cornea (**B**) compared to vehicle-treated cornea (**A**) indicates development of fibrosis in human cornea by NM (opacity is marked by a *blue arrow*). A notably increased αSMA (*green*; *yellow arrowhead*) and decreased TFAM (*red*; *white arrow*) fluorescence in the stroma of corneal tissue sections of fibrotic corneas (**D**) compared to normal cornea (**C**) suggests that the loss of TFAM is related to fibrosis development in human cornea ex vivo. Representative immunofluorescence images were taken at the central anterior stroma of the cornea. *Scale bar*: 50 µm. Isotypes controls for αSMA and TFAM are provided in Supplementary Figure S3.

Figure 7 shows expression of αSMA and TFAM in normal and fibrotic human corneas in ex vivo whole-cornea organ cultures (Figs. 7A, 7B) and corneal sections from them (Figs. 7C, 7D) analyzed at day 7. A notably blurred “letter A” observed in NM-treated corneas (Fig. 7B) compared with vehicle-treated corneas (Fig. 7A) confirmed development of fibrosis in the donor human corneas due to NM. Additionally, corneal sections of NM-treated fibrotic human cornea showed increased αSMA (Fig. 7D, green) and reduced TFAM (Fig. 7D, red) expression levels in stroma compared to vehicle-treated normal cornea (Fig. 7C, no αSMA and high TFAM expression). Immunofluorescence of isotype controls for αSMA and TFAM are presented in Supplementary Figure S3. These findings suggest that the loss of TFAM promotes fibrosis formation in human corneas ex vivo.

## Discussion

Excessive CMF formation and persistence in stroma after ocular injury have been shown to be a major cause of corneal fibrosis^39^; however, the role of mitochondrial defects in CMFs during corneal wound healing remains largely unknown. Coordinated expression of nuclear and mitochondrial proteins is paramount for proper mitochondrial functioning in CMFs. TFAM is a major regulator of mitochondrial gene expression and plays a critical role in maintaining structure, transcription, and replication of mtDNA*.* To the best of our knowledge, the current study is the first to demonstrate that mtDNA copies and mtDNA-to-nDNA ratios are significantly reduced in CMFs compared to CSFs, in addition to a time-dependent decrease in mtDNA and TFAM levels with a concurrent increase in mtROS levels and mitochondria numbers in transdifferentiation of CSFs to CMFs in vitro*.* The change in mtROS is significant, as mtDNA is highly susceptible to ROS damage because, unlike nDNA, mtDNA lacks histones, contains no introns, and lacks an efficient DNA repair mechanism.^40^ Similar to cardiac fibroblasts, this study determined that mtROS production corresponds to the loss of mtDNA.^14^^–^^16^ Further, to determine the role of TFAM in mtDNA regulation, the TFAM gene was silenced in human CSFs. TFAM silencing in human CSFs via gene editing via CRISPR/Cas9 caused a loss of mtDNA and increased αSMA expression de novo, suggesting that mtDNA plays a role in myofibroblast formation (Fig. 6).

An interesting finding of this study is that the loss of mtDNA is correlated with an increase in the number of mitochondria during CSF transdifferentiation to CMFs (Figs. 3, 4). Corneal mitophagy studies indicate that CMFs undergo mitochondrial fusion and fission to maintain increased numbers of mitochondria to accommodate increased ATP demand.^41^^,^^42^ However, mitochondria are known to induce mitochondrial dynamics including fusion, fission, and mitophagy to re-establish mtDNA quanity.^43^ This study indicates that, in addition to ATP demands, mitochondrial fusion and fission may increase to assist with mtDNA repair. Although mitochondria undergo fission to increase ATP and repair mtDNA, excessive fission has been determined to be detrimental to mitochondrial and cellular crosstalk.^44^^,^^45^ To assess if mitochondrial dynamics and nuclear crosstalk were altered in CMFs in our studies, mitochondrial mass was measured.^46^^,^^47^ The detection of a significant decrease in CMF mitochondrial mass (Fig. 2B) indicated that mitochondrial dynamics and nuclear crosstalk were affected during corneal fibrosis in vitro.

Along with a decrease in mitochondrial mass, this study identified a significant decrease in TFAM expression in CMFs compared to CSFs (Figs. 3B, 4B, 5B). To further elucidate the importance of mtDNA in CMFs, this study silenced TFAM in CSFs via gene editing and induced them to transdifferentiate into CMFs via TGFβ1 in vitro, and a significant decrease in mtDNA was observed (Fig. 6). These data suggest that TFAM regulates mtDNA transcription, mtDNA copy numbers, and mtROS production in CSFs and CMFs. This new finding in the cornea is consistent with cardiac and pulmonary fibrotic processes in which fibroblasts differentiate into myofibroblasts.^48^^–^^51^ Another key finding of this experiment was detection of the de novo expression of αSMA in TFAM-silenced CSFs grown in the absence of TGFβ1 (Fig. 6D). These data suggest that CSFs increased cell contraction, or transdifferentiation into CMFs is independent of TGFβ1.^41^^,^^43^^,^^52^ A potential cause of de novo αSMA expression is postulated to be because of recruitment of mtRNA polymerase and other transcription factors by TFAM required for the expression of oxidative phosphorylation proteins. The applicability of the conceptual findings from in vitro studies was verified using an ex vivo human cornea organ culture (Fig. 7). In this model, mustard gas exposure to human corneas ex vivo led to a significant corneal opacity and increased αSMA expression along with a significant decrease in TFAM (Fig. 7).

The implications of this study extend beyond corneal fibrosis, as mitochondria have been identified as a major contributor to many ocular pathologies including KSS, retinal pigmentary dystrophy, Leber hereditary optic neuropathy, chronic progressive external ophthalmoplegia (CPEO), and keratoconus.^22^^–^^24^ Additionally, this study provides a potential explanation for and a connection between the loss of mtDNA and irregular fibroblast activity, which may contribute to progressive stromal thinning in keratoconus patients. Moreover, the literature shows that variation in mitochondrial mass is a potential mechanism for guttae formation in Fuchs dystrophy patients. The present study found that alterations in mitochondrial mass are associated with loss of mtDNA. Hence, future studies are warranted to investigate how mtDNA alterations contribute to the pathogenesis of other ocular pathologies.

In summary, this study demonstrated that CMFs have mitochondria with significantly reduced mtDNA and TFAM. Further, it suggests that mtDNA regulates CMF activity and that preservation of TFAM potentially mitigates mtDNA damage during CMF formation.

## Supplementary Material

Supplement 1
