# Supplementary material for: Alterations in Mitochondrial DNA in Corneal Fibroblast and Myofibroblast Post Injury
Source: Invest Ophthalmol Vis Sci. 2026 Jan 16;67(1):36. doi: 10.1167/iovs.67.1.36 (PMC12831148; doi:10.1167/iovs.67.1.36)
Supplement: Supplement 1 [file iovs-67-1-36_s001.docx]

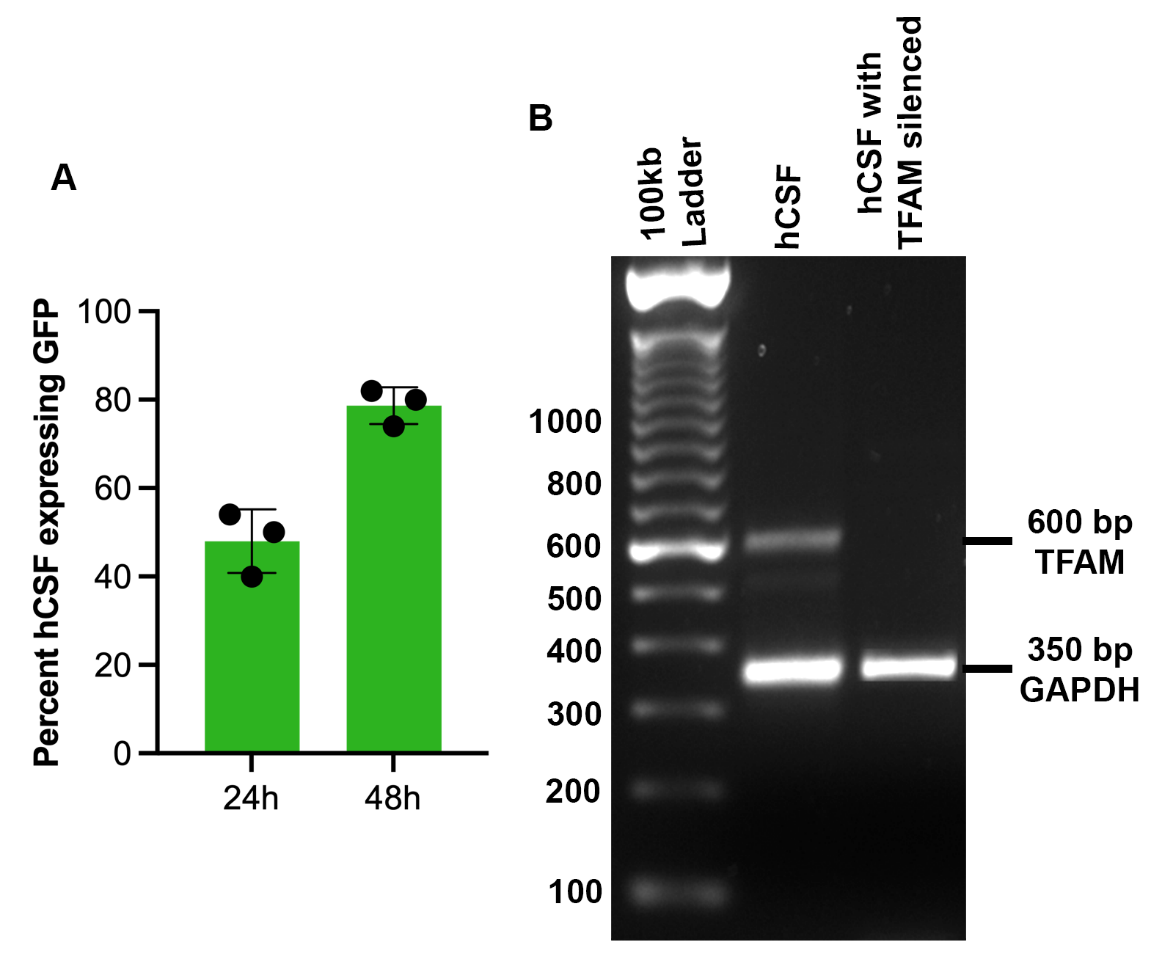


**Figure S1: TFAM silencing in hCSF treated with CRISPR/Cas9 TFAM KO plasmid**. The percent of hCSF that expressed GFP was calculated by comparing the number of GFP+DAPI+ cells to GFP-DAPI+ cells from six viewing fields at 100x magnification (A). Data shown is from three independent experiments with cells isolated form three separate donor corneas (n=3). PCR analysis shows TFAM was silenced in hCSF treated with TFAM KO plasmid (B). TFAM silencing was confirmed from two separate cultures.


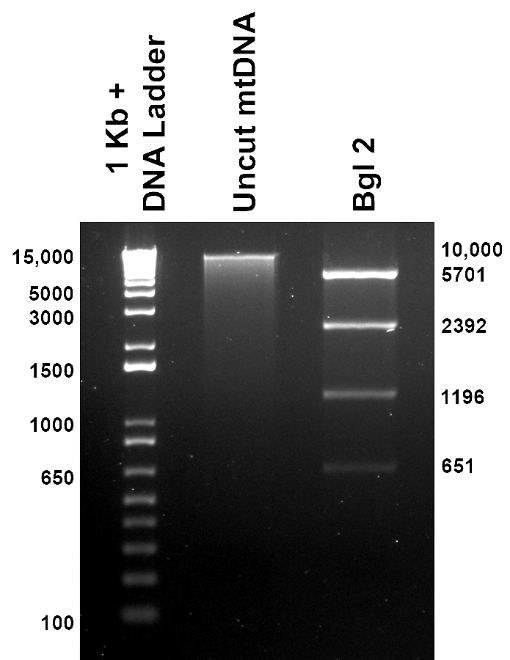


**Figure S2: Restriction enzyme mapping of mtDNA.** Bgl 2 restriction enzyme was used to confirm amplification of mtDNA. An expected four bands at 5701 bp, 2392 bp, 1196 bp, and 651 bp were detected in gel electrophoresis of DNA subjected to Bgl 2 endonuclease digestion. Restriction enzyme was performed on mtDNA amplification product from three independent experiments with cells isolated form three separate donor corneas (n=3).


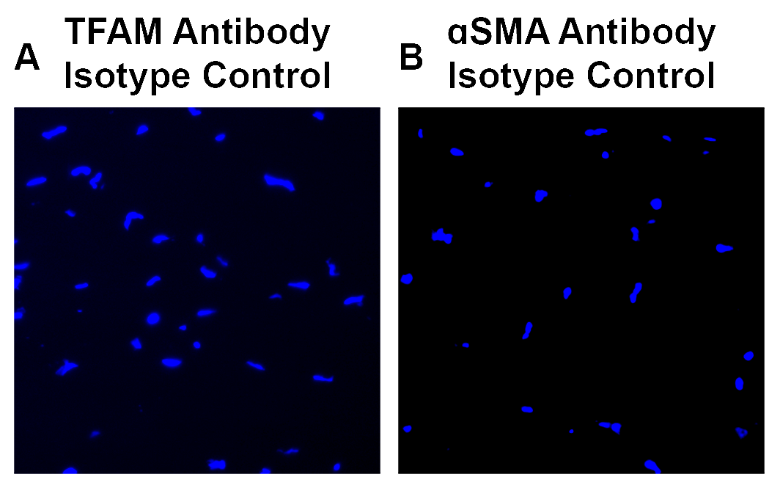


**Figure S3: Isotype controls for TFAM and ɑSMA antibodies.** Rabbit IgG and mouse IgGa kappa did not show any non-specific staining for TFAM and ɑSMA respectively. Representative immunofluorescence images were taken at the central anterior stroma of the cornea.
